# The Potential Therapeutic Effects of Testosterone on Sarcopenia: A Thorough Systematic Review

**DOI:** 10.7759/cureus.116239

**Published:** 2026-09-14

**Authors:** Bilal Khan, Rupanshu Rupanshu, Russaal S Mann, Isha Chopra, Abdullah Kilic, Ayushi Saxena, Pousette F Hamid

**Affiliations:** 1 Health Sciences, Queen's University, Kingston, CAN; 2 Internal Medicine, St. Martinus University Faculty of Medicine, Willemstad, CUW; 3 Internal Medicine, Vardhman Mahavir Medical College and Safdarjung Hospital, New Delhi, IND; 4 Anesthesiology, Mahatma Gandhi Medical College & Hospital, Jaipur, IND; 5 Internal Medicine, Hackensack University Medical Center, Hackensack, USA; 6 Medicine, California Institute of Behavioral Neurosciences & Psychology, Fairfield, USA; 7 Neurology, Ain Shams University, Cairo, EGY; 8 Neurology, California Institute of Behavioral Neurosciences & Psychology, Fairfield, USA

**Keywords:** role of testosterone, sarcopenia, sarcopenia management, testosterone (tt), therapeutic interventions

## Abstract

Sarcopenia is characterized by a progressive loss of skeletal muscle mass and function with aging, often resulting in functional decline and reduced independence. Although it primarily affects older adults, its development is multifactorial and may be influenced by various underlying factors. Testosterone, the primary endogenous male androgen, promotes skeletal muscle protein synthesis by activating pathways involved in muscle growth, cellular energy, and tissue repair. As testosterone levels decline with age, decreases in muscle strength and increases in frailty may occur, prompting investigation into testosterone replacement therapy (TRT) as a potential therapeutic intervention. This systematic review examines evidence from 15 studies comparing the effects of testosterone in patients with sarcopenia, with a focus on efficacy and functional improvements. Key outcomes include changes in lean body mass, strength performance, and physical function indicators. Our findings suggest that TRT can increase lean muscle mass and strength to a lesser extent, but improvements in mobility or daily function are inconsistent among population groups. Evidence regarding long-term safety and efficacy, as well as responses in the presence of other comorbidities, is limited. Current best practice recommendations emphasize exercise and a protein-rich diet as the primary approach for sarcopenia. This review aims to clarify the therapeutic role of testosterone in managing sarcopenia, highlight current limitations in the evidence base, and identify critical directions for future research.

## Introduction and background

Sarcopenia is the erosion of skeletal muscle as age progresses, generally causing functional decline that leads to a loss of independence [1]. This particularly affects older adults, although the diagnosis is broad and can have a wide range of underlying causes [1]. Testosterone, considered endogenously the main male androgen, promotes skeletal muscle protein synthesis and has a role in stimulating insulin-like growth factor 1 (IGF-1), which activates the PI3K-Akt pathway for protein synthesis, glucose uptake, and mitochondrial biogenesis [2]. After the age of 30, men lose approximately 1% of their testosterone annually, which correlates with diminished strength and increased frailty [3]. Currently, research is being conducted into whether testosterone replacement therapy (TRT) can mitigate sarcopenia. Some studies show that TRT can increase lean skeletal muscle mass and strength, although the practical benefits often vary [1,3,4]. Based on current evidence from the American College of Physicians, TRT is recommended as an intramuscular treatment for men with age-related low testosterone levels who have concerns about sexual function, but not for concerns about energy/vitality, physical function, or cognitive function due to low-certainty evidence [5]. Generally speaking, global guidelines regarding sarcopenia diagnosis and treatment encourage the use of the European Working Group on Sarcopenia in Older People and Foundation for the National Institutes of Health definitions and the prescription of resistance-based exercise supplemented with a protein-rich diet, respectively [6].

Despite efforts, several empirical knowledge gaps remain and persist across studies. Primarily, the long-term safety and efficacy of TRT in the sarcopenic population, and especially in older individuals with related and unrelated comorbidities, remain unclear [7,8]. Additionally, the majority of recommended interventions for sarcopenia attempt to improve environmental causes, such as activity levels and diet, and there have been few large-scale clinical trials that specifically target sarcopenia as a primary indicator [1]. Due to the multifactorial causes of sarcopenia, it is difficult to make a strict definition of it and to identify one key point of treatment, especially in terms of prescribed medication, resulting in treatments being indirect and symptomatically based. In this paper, we propose a systematic review of the present literature examining interventional testosterone use in adult populations with muscle mass or function used as indicators. Specifically, we are determining the effect of administered testosterone in patients with sarcopenia. The aim is to evaluate the strength and consistency of existing evidence, determine both safety and efficacy of TRT where possible, and identify areas in the research where further work is required.

## Review

Methods

Study Design and Reporting Guidelines

We conducted this systematic review in accordance with the PRISMA 2020 guidelines [9].

Eligibility Criteria

In this systematic review, we included papers published in the last five years, published in English with full text available, and based on human studies. A five-year publication window was selected to ensure that this review captured the most recent clinically relevant evidence and reduce the potential influence of older methodologies, diagnostic criteria, and therapeutic practices. Included articles contained information regarding how testosterone levels affect sarcopenia in patients. Papers published in non-English languages, lacking full-text articles, containing gray literature, and based on nonhuman studies were considered ineligible for this review and therefore excluded.

Search Strategy and Databases

The databases used to search for publications on the effects of testosterone on sarcopenia included PubMed Central, PubMed, ScienceDirect, Scopus, and Google Scholar. For a complete and thorough search, both Medical Subject Headings (MeSH) terms and keywords were used with Boolean operators. Specific keywords included “sarcopenia”, “testosterone”, and “therapeutic use of testosterone”. For simplification and concision, all relevant articles were imported into Zotero Citation Manager, and all duplicates were removed. Afterward, studies were manually screened based on the content of their titles and abstracts to remove irrelevant studies. The remaining papers were then reviewed on the basis of quality appraisal. The information about the search strategy is detailed in Table 1.

**Table 1:** Information on the search strategy used, including the specific filters.

Database	Keywords	Search strategy	Filters	Number of research papers
PubMed MeSH	Sarcopenia, testosterone, diagnosis, pathology, physiopathology, therapy, blood, deficiency, history, pharmacokinetics, pharmacology, physiology, therapeutic use	Sarcopenia OR ("Sarcopenia/diagnosis"[Majr] OR "Sarcopenia/pathology"[Majr] OR "Sarcopenia/physiopathology"[Majr] OR "Sarcopenia/therapy"[Majr]) AND Testosterone OR ("Testosterone/blood"[Majr] OR "Testosterone/deficiency"[Majr] OR "Testosterone/history"[Majr] OR "Testosterone/pharmacokinetics"[Majr] OR "Testosterone/pharmacology"[Majr] OR "Testosterone/physiology"[Majr] OR "Testosterone/therapeutic use"[Majr])	Last five years, humans, English language, free full text, subjects aged 19+	452
PubMed Central	Sarcopenia, testosterone, diagnosis, pathology, physiopathology, therapy, blood, deficiency, history, pharmacokinetics, pharmacology, physiology, therapeutic use	(Sarcopenia OR "Sarcopenia/diagnosis"[Majr] OR "Sarcopenia/pathology"[Majr] OR "Sarcopenia/physiopathology"[Majr] OR "Sarcopenia/therapy"[Majr]) AND (Testosterone OR "Testosterone/blood"[Majr] OR "Testosterone/deficiency"[Majr] OR "Testosterone/history"[Majr] OR "Testosterone/pharmacokinetics"[Majr] OR "Testosterone/pharmacology"[Majr] OR "Testosterone/physiology"[Majr] OR "Testosterone/therapeutic use"[Majr])	Last five years, humans, English language, free full text, subjects aged 19+	29
PubMed	Sarcopenia, testosterone	(sarcopenia[Title/Abstract]) AND (testosterone[Title/Abstract])	Last five years, humans, English language, free full text, subjects aged 19+	25
ScienceDirect	sarcopenia, testosterone	Sarcopenia, testosterone, sarcopenia[Title/Abstract/keyword], testosterone[Title/Abstract/keyword]	2021-2025, English, open access, and open archive	6
Scopus	sarcopenia, testosterone	Article title, Abstract, Keywords: testosterone AND sarcopenia	2021-2025, limited to article, limited to review, limited to conference paper, limited to final, excluded nonhuman, excluded animal, limited to sarcopenia, limited to testosterone, limited to English, limited to all open access	105
Google Scholar	sarcopenia, testosterone	allintitle: sarcopenia, testosterone	Since 2021	12

Because both primary studies and previously published reviews were eligible for inclusion, the reference lists of included reviews were compared with the primary studies identified in the search to identify potential overlap. Where the same primary study was represented both directly and within an included review, findings were interpreted cautiously to minimize double-counting of evidence.

Evidence Synthesis

Due to substantial heterogeneity in study designs, participant characteristics, testosterone exposure and treatment regimens, comparators, follow-up periods, and outcome measures, a quantitative meta-analysis was not considered appropriate.

No pooled effect estimates or statistical tests of between-study heterogeneity were performed because a quantitative meta-analysis was not conducted. Similarly, I² statistics and formal statistical tests for publication bias were not applicable. Individual p-values and confidence intervals reported by the original studies were retained where relevant but were not statistically combined or reanalyzed.

Instead, findings were synthesized narratively and grouped according to study design and primary outcome domains. Evidence was organized into three broad domains: (1) associations between endogenous testosterone and body composition or muscle function; (2) effects of testosterone supplementation on muscle mass and functional outcomes; and (3) evidence regarding multimodal interventions combining testosterone with resistance exercise and/or nutritional support.

Findings from randomized controlled trials (RCTs) were considered separately from observational studies and narrative or systematic reviews to distinguish evidence regarding treatment effects from evidence regarding associations. Particular emphasis was placed on functional outcomes, including muscle strength and physical performance, rather than changes in lean muscle mass alone. Where studies reported inconsistent findings, differences in population characteristics, intervention protocols, outcome measures, and study duration were considered when interpreting the results.

Results

Study Selection

The literature search was conducted from June 1, 2021, to June 1, 2025, and produced a total of 629 references retrieved from six databases: PubMed, PubMed Central, PubMed MeSH, ScienceDirect, Scopus, and Google Scholar. After removing 84 duplicate references, there were 545 unique records. The titles and abstracts of the remaining records were then screened, and 483 records were deemed irrelevant, leaving 62 potentially eligible references. After full-text screening of the remaining records, 45 were excluded, leaving a final selection of 17 references.

Following removal of duplicate records, two reviewers independently screened titles and abstracts according to the predefined eligibility criteria. Potentially eligible articles underwent independent full-text assessment. Data were independently extracted using a standardized approach, including study design, population, sample size, testosterone exposure or intervention, comparator, and relevant sarcopenia-related outcomes. Disagreements during screening or data extraction were resolved through discussion and consensus, with a third reviewer consulted when consensus could not be reached.

Afterward, a quality assessment was performed using dedicated instruments for each study type, leading to the inclusion of 15 high-quality studies with a score of 70% or more. The final research study consisted of one systematic review, six narrative reviews, three RCTs, and four cross-sectional studies. Figure 1 displays the flow diagram showing the search and selection process.

**Figure 1:**
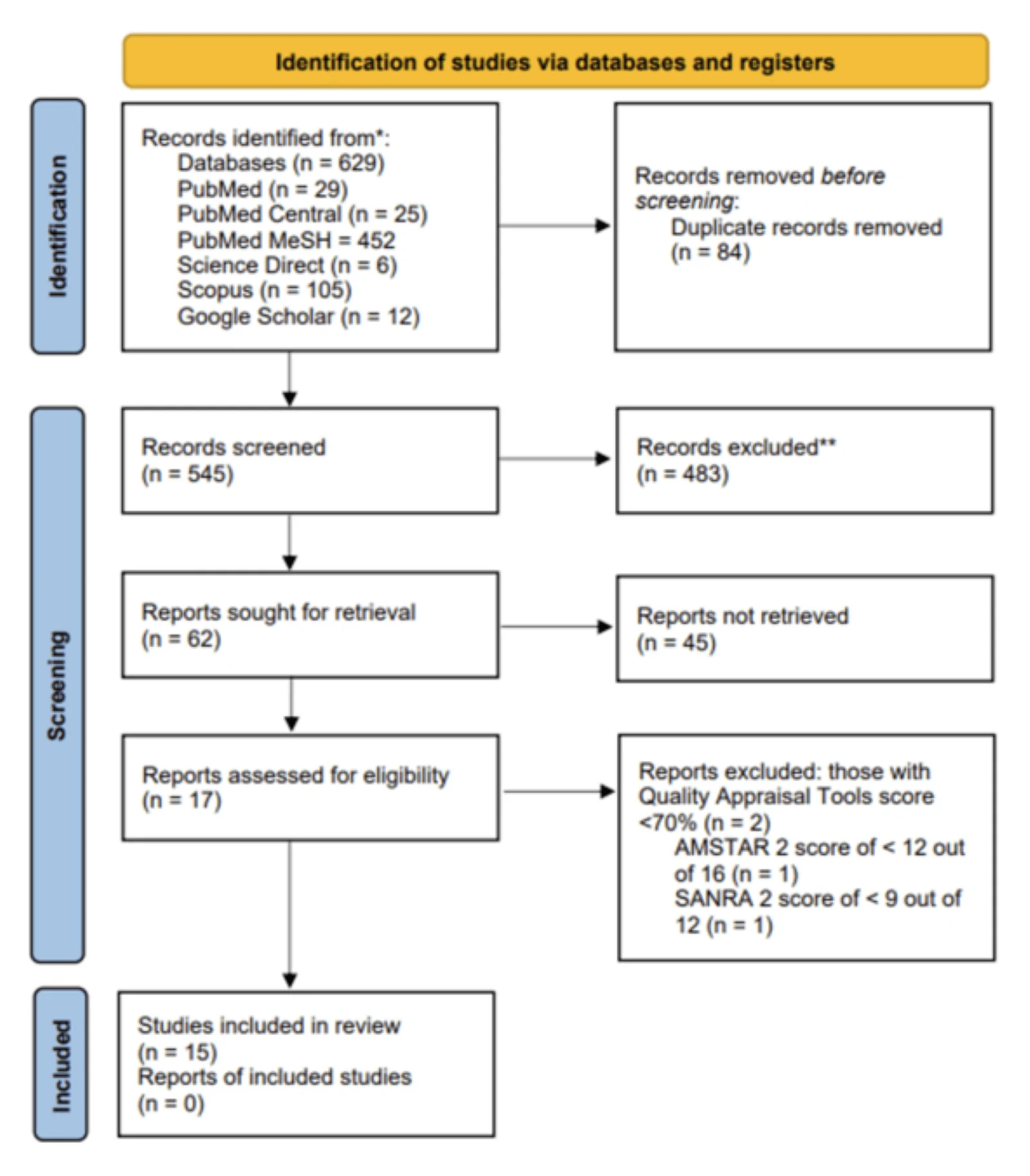
PRISMA flowchart. AMSTAR 2: Assessment of Multiple Systematic Reviews 2; SANRA 2: Scale for the Assessment of Narrative Review Articles 2

Study Characteristics and Risk of Bias

A thorough summary and examination of the quality assessment process, alongside the specific tools used to evaluate the articles for this review, is presented in Table 2. Although all included studies met the predefined quality threshold, methodological limitations remained across the evidence base. In particular, the cross-sectional studies were limited in their ability to establish temporal or causal relationships, while the RCTs were generally characterized by small sample sizes and relatively short follow-up periods. The inclusion of reviews also introduced potential overlap with primary studies. These factors were considered when interpreting the overall evidence. This systematic review was not prospectively registered in PROSPERO or another systematic review registry.

**Table 2:** Detailed analysis of the quality assessment procedure and the methods and tools used to analyze the reviewed articles. AMSTAR 2: Assessment of Multiple Systematic Reviews 2; AXIS: Appraisal tool for Cross-Sectional Studies; CCRBT: Cochrane Collaboration Risk of Bias Tool; SANRA 2: Scale for the Assessment of Narrative Review Articles 2

Quality assessment tool	Type of study	Total score	Accepted score (>70%)	Number of accepted studies (n)
AMSTAR 2	Systematic review	9	7	1 (Welch et al. [10])
SANRA 2	Narrative review	12	9	6 (Chrysavgis et al. [6], Huang and Wang [8], Gungor et al. [11], Kim et al. [12], Shigehara et al. [13], Dos Santos et al. [14])
CCRBT	RCTs	7	5	3 (Chasland et al. [4], Rhee et al. [15], Midttun et al. [16])
AXIS	Cross-sectional studies	20	14	4 (Guligowska et al. [1], Ye et al. [17], Liu et al. [18], Schluessel et al. [19])

A summary of the main characteristics of the systematic reviews included in the analysis is presented in Table 3.

**Table 3:** Major features of the key features of the systematic reviews used in the study. NR: narrative review; RCT: randomized controlled trial

Author	Study type	Inclusion criteria	Outcome
Chrysavgis et al. (2024) [6]	Traditional review	NR	It was concluded that chronic liver disease, hypogonadism, and low testosterone are key contributors to sarcopenia and frailty, and that testosterone supplementation offers anabolic improvements but does not demonstrate a benefit in survival because of the limited quality and strength of evidence.
Huang and Wang (2021) [8]	Traditional review	NR	It was found that testosterone has clear anabolic effects on muscle mass and strength, but evidence for functional improvement and long-term health outcomes is inconsistent and underexplored.
Welch et al. (2021) [10]	Systematic review	RCTs and quasi-RCTs studying hospitalized patients ≤65 years old with pre- and post-intervention measurements of muscle quantity, strength, or physical performance were available.	The review indicated that prescriptions of physical activity led to greater functional improvement but were limited by frailty. By comparison, nutrition also had small effects on strength and mass. Testosterone and other anabolic agents had inconclusive, limited, and mixed results. Note that the mean age was 79.1 years, and participants were 59% female.
Gungor et al. (2021) [11]	Traditional review	NR	The review showed that testosterone alone did not significantly improve leg strength in isolation but reduced fat composition. The multimodal intervention (testosterone, resistance training, and nutrition) showed clinically relevant improvements.
Kim et al. (2023) [12]	Traditional review	NR	The review indicated that testosterone therapy as a hormone-based intervention for sarcopenic obesity can reduce fat mass and improve muscle strength, but more controlled trials are necessary to validate its potential.
Shigehara et al. (2022) [13]	Traditional review	NR	The review observed that low testosterone is strongly linked to sarcopenia and frailty and that testosterone supplementation appears promising. However, functional and quality-of-life improvements are not well supported, especially in long-term multimodal interventions.
Dos Santos and Storer (2022) [14]	Traditional review	NR	The review showed that testosterone presents as a potential therapeutic agent for patients with low testosterone levels due to clear anabolic effects on muscle volume and strength, but its impact on functional improvement is still uncertain.

A summary of the main characteristics of the RCTs included in the analysis is presented in Table 4.

**Table 4:** Major features of RCTs incorporated into the study. DXA: dual-energy X-ray absorptiometry; eGFR: estimated glomerular filtration rate; LDL: low-density lipoprotein; RCT: randomized controlled trial

Author	Study type	Inclusion criteria	Sample size (n = 270)	Outcome
Chasland et al. (2021) [4]	RCT	Men aged 50-70 years with serum testosterone of 6-14 nmol/L, cholesterol ≤7 mmol/L or LDL ≤4 mmol/L, eGFR ≤45 mL/min, and no evidence of liver disease.	80	This study’s results indicated that testosterone can augment lean body mass (p = 0.015) but is insufficient for significant strength improvements without exercise supplementation (p > 0.05), measured with DXA.
Rhee et al. (2021) [15]	RCT	Adults aged 18-80 years who were commencing or receiving androgen deprivation therapy.	42	The study showed that liver-targeted testosterone therapy has potential for use as a safeguard against sarcopenia (p < 0.04), measured with a 30-second chair stand test, and osteopenia (p < 0.02), measured with DXA, during androgen deprivation therapy.
Midttun et al. (2024) [16]	RCT	Males ≤70 years old with an increased risk of falling, testosterone <13 nmol/L, and self-reported or tested weakness, reduced walking ability, gait instability, or repeated falls.	148	The study found that testosterone by itself improved body composition (p < 0.05) but did not enhance functional strength (p > 0.05), measured with DXA and the 30-second chair stand test, respectively. The combination of testosterone therapy with resistance training and nutrition led to relevant and significant improvements, however (p < 0.01).

A summary of the main characteristics of the cross-sectional studies included in the analysis is presented in Table 5.

**Table 5:** Significant aspects of cross-sectional studies taken into account in the analysis. NHANES: National Health and Nutrition Examination Survey

Author	Study type	Inclusion criteria	Sample size	Outcome
Guligowska et al. (2021) [1]	Cross-sectional study	Participants aged ≥75 years with functional independence, good verbal and logical communication, and no signs of malnutrition	100	Higher endogenous testosterone correlated with better body composition in terms of lower fat mass in men (p < 0.05), and gonadotropins such as follicle-stimulating hormone and luteinizing hormone showed strong positive relationships with muscle mass, strength, and performance (p < 0.05).
Ye et al. (2021) [17]	Cross-sectional study	Men aged 20-59 years in the NHANES survey database, with data collected from 2011 to 2016	3,875	The study indicated that serum testosterone was positively associated with lean mass (p < 0.001) and negatively associated with fat mass (p < 0.001) and was consistently linked to higher bone density (p < 0.001), even after adjustment for BMI and other covariates.
Liu et al. (2025) [18]	Cross-sectional study	Participants aged 20-60 years in the NHANES dataset from 2011 to 2014 with available LE8 and covariate data	4,062	The study showed that higher testosterone levels correlated with a lower risk of having low muscle mass and that testosterone mediated 15-16% of the protective association between cardiovascular health and muscle mass (95% CI: -0.0007, -0.0003).
Schluessel et al. (2024) [19]	Cross-sectional study	Patients older than 65 years with available data on body composition, bone mineral density, handgrip strength, and endocrinological laboratory measurements	167	The study determined that there was a correlation between hypogonadism and clinical sarcopenia (p < 0.05) and that lower testosterone levels were correlated with reduced handgrip strength (p < 0.05).

Discussion

Summary of Main Findings

This systematic review collected evidence from 15 studies, including RCTs, narrative reviews, systematic reviews, and cross-sectional studies, to determine a relationship between testosterone and sarcopenia outcomes. Generally, low endogenous levels of serum testosterone consistently correlate with low lean mass, high fat mass, and reduced functional strength as measured by grip strength and other performance-based metrics. Based on some trials, testosterone supplementation can lead to improvements in lean muscle mass, but increases in functional strength are inconclusive unless it is used in a multimodal intervention with an exercise and nutrition plan. Beyond body composition changes, testosterone monotherapy rarely improves strength or performance.

Older populations, especially inpatients, indicate a high prevalence of hypogonadism and sarcopenia with mutually reinforcing factors. The role of testosterone is inherently complicated, and while the functional impact is context-dependent, the anabolic signaling pathway is biologically plausible and measurable. Safety data regarding testosterone are limited in size and duration, but what currently stands reflects good tolerability with minor side effects and some cardiovascular concerns.

Biological Plausibility and Mechanistic Context

Testosterone acts anabolically through androgen receptor-mediated pathways, IGF-1/Akt/mTOR signaling, satellite cell activation, and inhibition of catabolic factors like myostatin [15]. Endogenous testosterone prompts muscle fiber hypertrophy, especially in type I and II fibers, and it may enhance mitochondrial function while reducing inflammatory cytokines like IL-6 and TNF-alpha [4]. These mechanisms support the cross-sectional and observational associations found in National Health and Nutrition Examination Survey-derived studies, in which higher testosterone levels are correlated with higher lean mass, lower fat mass, and higher bone mineral density, even with adjustments for covariates such as BMI and age [20].

Endogenous Testosterone and Sarcopenia Risk

There have been several cross-sectional studies that have, across the board, demonstrated that lower endogenous testosterone levels are significantly associated with adverse relationships in body composition and function indicators (e.g., decreased strength) [8,15,21]. These associations frequently persisted even after adjusting for covariates and potential confounders, strongly suggesting an independent influence on sarcopenia risk. Given the close relationship between sarcopenia and physical frailty, particularly through shared characteristics such as reduced muscle strength and physical performance, testosterone deficiency may also contribute to the development of frailty indirectly through its effects on muscle health. This relationship is most clearly evident and prevalent in the geriatric inpatient population, where low testosterone worked as a predictor for frailty [12]. However, given the predominantly cross-sectional nature of this evidence, these findings demonstrate association rather than establishing a causal relationship between testosterone deficiency, sarcopenia, and frailty.

Testosterone Supplementation and Muscle Outcomes

An RCT conducted by Chasland et al. found that testosterone led to increases in lean muscle mass, as the testosterone + no exercise group had an average increase of 0.7 kg from baseline after 12 weeks, with increases of 0.5 kg and 0.2 kg in the legs and arms, respectively [4]. Comparatively, the exercise + placebo group had increases of 0.7, 0.1, and 0.3 kg in the lean muscle mass of the overall body, legs, and arms, respectively [18]. This is consistent with other trials that demonstrate similar findings of testosterone supplementation/therapy increasing lean muscle mass [20,22,23].

Testosterone therapy, despite its effect on body composition and lean muscle mass, appears not to have a clinically significant effect on increasing functional strength. Only when combined with muscle training does testosterone appear to have any important improvement, as demonstrated by Midttun et al. in their RCT [16]. Welch et al. were not able to come to a conclusive statement on the effect of testosterone on leg muscle strength due to a lack of data but implied that any effect testosterone supplementation would have would be minimal and limited at best [10].

Safety, Tolerability, and Adverse Effects

While the anabolic effects show some promise, testosterone therapy is unfortunately not risk-free. The U.S. FDA has highlighted potential increased cardiovascular events and stroke associated with testosterone, particularly in older men with naturally low endogenous serum testosterone levels as a result of aging [3]. Some common side effects include increased hematocrit, acne, exacerbation of sleep apnea, and, in rare cases, pulmonary oil microembolisms [13]. Generally, however, the long-term safety of androgen therapy remains understudied, as none of the reviewed trials were beyond 20 weeks. As a guideline, androgen therapy should be tailored individually with monitoring in patients, especially those with cardiovascular, liver, kidney, or prostate disease.

Context Within Current Guidelines and Therapeutic Landscape

Currently, the U.S. FDA has not formally approved any pharmacotherapies for sarcopenia [3], and all present pharmaceutical efforts are considered experimental. Guidelines from the European Working Group on Sarcopenia in Older People and the Asian Working Group for Sarcopenia both strongly recommend resistance training and appropriate nutrition as the first interventions for muscle loss management [17,24]. Current emerging alternatives like SARMs and novel anabolics have also shown a similar issue of increasing lean muscle mass without functional enhancement, and as a result, regulatory approval has been denied [25].

Testosterone supplementation therapy is presently recognized as a treatment for symptomatic hypogonadism, but modern guidelines err on the side of caution, i.e., to reserve TRT for men with clinical symptoms and confirmed low testosterone [8]. Recent reviews call for longer randomized trials with further follow-up post-completion and detailed looks at functional endpoints and safety standards [8,18].

Integration of Multimodal Interventions

The evidence firmly points to the strongest therapeutic effect coming from combining testosterone therapy with resistance exercise and nutritional support. The trials by Chasland et al. and Midttun et al. both displayed additive gains in lean mass and functional performance that exceeded either testosterone or exercise alone [4,16]. From this perspective, testosterone can serve as a muscle-preserving complement by raising anabolic thresholds in those with sarcopenia, and especially those who have hypogonadism, but on its own, it will not replace the current standards of exercise or nutritional optimization.

Heterogeneity and Limitations Across Studies

The study populations varied widely across the selected studies, and as a result, the variability in baseline testosterone, comorbidity burden, and status of physical functionality or frailty limits generalizability. In addition, the testosterone formulations differed between oral and intramuscular routes of administration, dosing plans, target serum levels, and the duration of each trial. Comparators were typically either a placebo or resistance training, and infrequently nutrition or other androgen alternatives. There was also minimal follow-up post-study completion, making it difficult to make long-term inferences.

The functional outcomes relating to testosterone were also often secondary and underpowered due to small sample sizes. Body composition data were easier to measure and more sensitive to change, but the same cannot be said for evidence relating to functional strength, gait improvement, or overall physical improvement. A fair number of trials also lacked formal power calculations for the relevant functional endpoints.

The cross-sectional studies also only inform association, not causation. The clinical trials were generally small, with approximately 20-40 patients per arm, short-term, and had small, nondiverse populations. Very few addressed long-term endpoints such as fall rates, hospitalizations, fractures, or mortality.

Research Gaps and Future Directions

Future studies should focus more on the functional aspect of testosterone on sarcopenia rather than having the increase in lean muscle mass be the focus. Other long-term outcomes to look out for would include fall rates, fractures, mobility, quality-of-life improvements, and mortality. In addition, testosterone thresholds should be more clearly defined to determine at which point muscle decline accelerates and becomes critical and indicative of sarcopenia. The dose-response relationship should also be significantly evaluated, in particular with the elderly and frail.

Clinical trials should further broaden the range of treatment arms, not only to compare testosterone and exercise therapies but also to evaluate alternatives to testosterone and, especially, combination therapies involving testosterone supplementation and other treatments. More safety surveillance is also necessary to monitor the long-term effects on the cardiovascular, hematological, and prostate systems to ensure that testosterone is a reasonably safe treatment. Further, biological markers such as genetic markers, inflammatory mediators, and gonadotropins, especially in those in old age, should also be investigated due to how they may affect responsiveness.

Strengths and Limitations

A major strength is the inclusion of several diverse study designs that looked at testosterone and sarcopenia across a spectrum of populations and functional status. The review also explicitly focuses on testosterone-related outcomes and synthesizes compositional, functional, and mechanistic data so as not to lose focus. However, this review is limited by the heterogeneity of the studies and the absence of quantitative meta-analysis based on functional endpoints. Furthermore, the inclusion of narrative and systematic reviews alongside primary RCTs and observational studies introduces a potential risk of overlap between studies, whereby the same primary study may be represented both directly and through an included review. Although efforts were made to identify and avoid duplication where possible, complete exclusion of this overlap cannot be guaranteed. Broad inclusion of studies may also mix low- and high-quality evidence, and publication bias cannot be excluded either. In addition, the absence of prospective protocol registration may limit methodological transparency and increase the possibility of deviations from the initially intended review methodology. Despite these limitations, however, this paper proposes a cohesive narrative around the current role of testosterone in sarcopenia.

## Conclusions

This systematic review affirms current evidence on the relationship between serum testosterone levels and sarcopenia, especially in aging populations. It is consistently confirmed that endogenous low testosterone, whether due to age-related decline or symptomatic hypogonadism, is a strong correlate of sarcopenic risk, which includes reduced lean muscle mass, decreased muscle strength, and lower physical performance. This attests to how testosterone plays a critical anabolic role in maintaining muscle health, especially in older individuals. Evidence from several studies asserts that testosterone supplementation or TRT reliably increases lean muscle mass. It was also found that testosterone can improve physical functional performance in those with sarcopenia when combined with a resistance training regimen and appropriate nutrition. Unfortunately, testosterone monotherapy is unlikely to yield functional gains alone, but long-term results are still inconclusive due to a significant lack of data.

Despite some promising findings, the long-term safety profile of testosterone administration, especially in older adults, remains a concern. Side effects are generally mild, if present at all, but larger and longer-term studies are needed to assess the full long-term extent of the cardiovascular, prostate, and metabolic risks. Additionally, the heterogeneity in study design, diagnostic criteria, and intervention protocols limits the generalizability of current findings. To conclude, testosterone represents a potential modifiable hormonal factor to manage sarcopenia, with potential therapeutic benefits when combined with lifestyle-type interventions such as exercise and nutritional plans. Further research is necessary to determine the optimal dosing, duration, and patient selection criteria of testosterone therapy to safely integrate it into modern sarcopenia treatment guidelines.
